# Comparative Analysis of Stress Levels Among Undergraduate Students at St. John's College, Agra

**DOI:** 10.7759/cureus.67452

**Published:** 2024-08-22

**Authors:** Utkarsh Shrivastava, Aditya Thakur, Tej Pratap Singh, Jagmohan Singh Dhakar, Sanjay Jain, Ambika Agrawal, Hariom Pachori, Shubhangi Thakur

**Affiliations:** 1 Psychiatry, Jawaharlal Nehru Medical College, Datta Meghe Institute of Higher Education and Research, Wardha, IND; 2 Community Medicine, Netaji Subhash Chandra Bose Medical College, Jabalpur, IND; 3 Community Medicine, Sukh Sagar Medical College and Hospital, Jabalpur, IND; 4 Community Medicine, Virendra Kumar Sakhlecha Government Medical College, Neemuch, IND; 5 Statistics, St. John's College, Agra, IND; 6 Statistics, Central Institute of Psychiatry, Ranchi, IND; 7 Psychiatry, Netaji Subhash Chandra Bose Medical College, Jabalpur, IND

**Keywords:** sociodemographic factors, background, stress management, cross-sectional study, perceived stress scale (pss), undergraduate students

## Abstract

Background

Stress is prevalent among college students, impacting their mental health and academic performance. Understanding the distribution and determinants of stress levels in students is crucial for developing effective interventions. This study aims to assess the prevalence of stress and its association with sociodemographic factors among undergraduate students at St. John's College, Agra, India.

Materials and methods

A descriptive cross-sectional study was conducted between August 1, 2023, and December 31, 2023, involving 160 undergraduate students from B.A., B. Com., and B.Sc. programs. Students were selected using a stratified random sampling technique. Stress levels were measured using the Perceived Stress Scale (PSS), which classifies low, moderate, and high levels. Data were collected through a validated, semi-structured questionnaire administered via Google Forms. Statistical analysis was performed using IBM SPSS Statistics for Windows, Version 29 (Released 2023; IBM Corp., Armonk, New York), with descriptive statistics and chi-square tests used to examine associations between stress levels and sociodemographic variables.

Results

The study found that 103 (64.4%) of students experienced moderate stress, 34 (21.3%) reported high perceived stress, and 23 (14.4%) had low stress. A significant association was observed between stress levels and gender (p = 0.022), with female students more likely to experience high stress. Additionally, urban students reported higher stress levels than their rural counterparts (p = 0.012). However, no significant differences in stress levels were found across different courses.

Conclusion

The study reveals a substantial prevalence of moderate to high stress among college students, particularly among females and those from urban areas. These findings suggest the need for targeted stress management interventions to support student well-being. Further research is recommended to explore the underlying causes of stress and develop comprehensive stress reduction strategies in the student population.

## Introduction

Stress among university students has emerged as a critical concern in recent years due to its profound impact on mental health and academic performance [1]. Significant changes and challenges, including increased academic demands, social adjustments, and personal responsibilities, often mark the transition to higher education [2]. These factors contribute to elevated stress levels, which can adversely affect students' overall well-being and their ability to succeed academically [1,3]. Research has consistently demonstrated that university students are susceptible to high levels of stress, which can manifest as anxiety, depression, and a range of physical health issues [4,5].

The Perceived Stress Scale (PSS), developed by Cohen et al. in 1983, is one of the most widely used tools for assessing individual stress levels. This scale measures how unpredictable, uncontrollable, and overloaded respondents find their lives, and it has been validated across diverse populations [6]. Studies utilizing the PSS have revealed that moderate to high levels of stress are prevalent among students, with significant implications for their mental health and academic outcomes [7]. For example, a study conducted in India found that 57.9% of students reported experiencing significant stress, aligning with global findings that indicate high stress levels among student populations [8,9].

Sociodemographic factors play a crucial role in shaping students' stress experiences. Gender differences in stress perception are notable, with female students often reporting higher stress levels compared to male students [10]. This gender disparity may be attributed to various factors, including societal expectations, academic pressures, and personal coping mechanisms. Additionally, the area of residence, whether urban or rural, can influence stress levels due to differences in access to resources and support systems. Urban students may experience higher stress due to the fast-paced environment and greater academic competition, while rural students might face stress related to isolation and limited access to educational resources [10].

Understanding the interplay between these sociodemographic factors and stress is essential for developing effective interventions. By addressing the specific needs of different student groups, institutions can implement targeted strategies to support students in managing stress and enhancing their academic performance. This approach helps mitigate the negative effects of stress and promotes a healthier and more productive academic environment.

## Materials and methods

Study design and setting

This descriptive cross-sectional study was conducted at St. John's College, Agra, over five months, from August 1, 2023, to December 31, 2023. The study aimed to assess the stress levels among undergraduate students across various academic programs within the college.

Study population and sample size

The study population included undergraduate students in St. John's College's B.Sc., B. Com., and B.A. programs. A total of 160 students were included in the study, selected through a stratified random sampling technique. The sample size was determined based on a previous study that reported a 57.9% prevalence of stress among students and an absolute error of 8%. Using Cochran's formula, the minimum required sample size was calculated to be 145, rounded to 160 to account for 10% non-responders. The sample was proportionally divided among the faculties, with 40 students from B.A., 40 from B. Com., and 80 from B.Sc.

Inclusion and exclusion criteria

The study included students who voluntarily participated and were present during the study period. Students absent during the data collection phase were excluded from the study.

Data collection and study tools

Data were collected using a semi-structured questionnaire developed in Google Forms. The questionnaire was pretested and validated before distribution. The survey consisted of two sections: one capturing demographic data and the other assessing stress levels using the PSS [11]. The PSS, a widely recognized tool for measuring perceived stress, consists of 10 items with scores ranging from 0 to 40. Higher scores indicate higher levels of perceived stress, categorized as low (0-13), moderate (14-26), or high (27-40). The questionnaire link was emailed to 160 students who completed the form.

Ethical considerations and data analysis

Ethical clearance was obtained from the Institutional Ethics Committee. Anonymity was maintained by excluding any identifying information from the questionnaire. Informed consent was obtained electronically via the Google Form. Data were entered into Microsoft Excel (Microsoft Corporation, Redmond, Washington) and analyzed using IBM SPSS Statistics for Windows, Version 29 (Released 2023; IBM Corp., Armonk, New York). Descriptive statistics, including means, standard deviations, and percentages, were used to summarize the data. The three stress levels (low, moderate, high) were combined into a single category, "presence of stress," for analysis. Pearson's chi-square test for trend and odds ratios was employed to evaluate the association between stress levels and various study variables.

## Results

Table 1 presents the demographic characteristics of the study participants.

**Table 1:** Demographic characteristics of the study participants

Demographic Variables		N	%
Age group	18-19	89	55.63
	20-21	58	36.25
	>22	13	8.13
Gender	Female	80	50.00
	Male	80	50.00
Residence	Rural	30	18.75
	Urban	130	81.25
Father’s education	Up to higher secondary	48	30.00
	Graduate	71	44.38
	Postgraduate	35	21.88
	Illiterate	51	31.88
Mother’s education	Up to higher secondary	69	43.13
	Graduate	58	36.25
	Postgraduate	20	12.50
	Illiterate	13	8.13
Mother’s occupation	Housewife	146	91.25
	Working women	14	8.75
No. of friends	1 to 2	40	25.00
	3 to 4	36	22.50
	5 and above	84	52.50
No. of family members	1 to 2	1	0.63
	3 to 4	41	25.63
	5 and above	118	73.75
Religion	Hindu	156	97.50
	Muslim	3	1.88
	Other	1	0.63
Family monthly income	10,000-20,000	1	0.63
	30,000-40,000	41	25.63
	>50,000	118	73.75
Total		160	100.00

Table 2 illustrates the distribution of stress levels among the 160 students surveyed. The majority of students (103, 64.4%) reported moderate stress levels, followed by 34 (21.3%) experiencing high perceived stress and 23 (14.4%) reporting low stress. This distribution indicates that most students are experiencing a significant stress level, with a notable proportion facing high stress.

**Table 2:** Distribution and student stress management categories

Levels of Stress	Frequency (N)	Percentage (%)
Low stress	23	14.4
Moderate stress	103	64.4
High perceived stress	34	21.3
Total	160	100.0

Table 3 compares stress levels across courses (B.A., B. Com., and B.Sc.). Students in the B.Sc. program exhibited the highest proportion of low stress (69.6%) compared to B.A. and B. Com. students. However, high perceived stress was most prevalent among B. Com. students (35.3%). Although the differences in stress levels across courses are evident, the p-value of 0.138 suggests that these differences are not statistically significant.

**Table 3:** Stress level comparison in different courses

Course	Stress Level			p-value
	Low stress (N=23)	Moderate stress (N=103)	High perceived stress (N=34)	
BA	3 (13.0%)	27 (26.2%)	10 (29.4%)	0.138
BCom	4 (17.4%)	24 (23.3%)	12 (35.3%)	
BSc	16 (69.6%)	52 (50.5%)	12 (35.3%)	
Total	23 (100%)	103 (100%)	34 (100%)	

Table 4 examines the association between stress levels and various sociodemographic variables. Significant associations were observed for gender (p = 0.022) and area of residence (p = 0.012). Female students and those from urban areas were more likely to report high perceived stress. Other variables, such as age group, parental education, mother's occupation, number of friends, family members, religion, and economic status, did not show statistically significant associations with stress levels, suggesting that these factors may not strongly influence stress among the students studied.

**Table 4:** Association of stress level and different sociodemographic variables *Statistically significant, Fisher's exact test

Variables		Stress Level			p-value
		Low stress (N=23)	Moderate stress (N=103)	High perceived stress (N=34)	
Age group	18-19	16 (69.6%)	52 (50.5%)	21 (61.8%)	0.422
	20-21	5 (21.7%)	42 (40.8%)	11 (32.4%)	
	>22	2 (8.7%)	9 (8.7%)	2 (5.9%)	
Gender	Female	9 (39.1%)	47 (45.6%)	24 (70.6%)	0.022*
	Male	14 (60.9%)	56 (54.4%)	10 (29.4%)	
Residence	Rural	3 (13%)	26 (25.2%)	1 (2.9%)	0.012*
	Urban	20 (87%)	77 (74.8%)	33 (97.1%)	
Father's education	Up to higher secondary	6 (26.1%)	35 (34%)	7 (20.6%)	0.263
	Graduate	11 (47.8%)	47 (45.6%)	13 (38.2%)	
	Postgraduate	6 (26.1%)	17 (16.5%)	12 (35.3%)	
	Illiterate	0 (0.0%)	49 (47.6%)	2 (5.9%)	
Mother's education	Up to higher secondary	9 (39.1%)	47 (45.6%)	13 (38.2%)	0.565
	Graduate	11 (47.8%)	33 (32%)	14 (41.2%)	
	Postgraduate	3 (13%)	12 (11.7%)	5 (14.7%)	
	Illiterate	0 (0%)	11 (10.7%)	2 (5.9%)	
Mother's occupation	Housewife	22 (95.7%)	94 (91.3%)	30 (88.2%)	0.621
	Working women	1 (4.3%)	9 (8.7%)	4 (11.8%)	
No. of friends	1 to 2	7 (30.4%)	25 (24.3%)	8 (23.5%)	0.714
	3 to 4	7 (30.4%)	22 (21.4%)	7 (20.6%)	
	5 and above	9 (39.1%)	56 (54.4%)	19 (55.9%)	
No. of family members	1 to 2	0 (0.0%)	1 (1.0%)	0 (0.0%)	0.894
	3 to 4	7 (30.4%)	26 (25.2%)	8 (23.5%)	
	5 and above	16 (69.6%)	76 (73.8%)	26 (76.5%)	
Religion	Hindu	23 (100.0%)	100 (97.1%)	33 (97.1%)	1
	Muslim	0 (0.0%)	2 (1.9%)	1 (2.9%)	
	Other	0 (0.0%)	1 (1.0%)	0 (0.0%)	
Family monthly income	10,000-20,000	0 (0.0%)	1 (1.0%)	0 (0.0%)	0.485
	30,000-40,000	7 (30.4%)	26 (25.2%)	8 (23.5%)	
	>50,000	16 (69.6%)	76 (73.8%)	26 (76.5%)	

## Discussion

This study aimed to assess stress levels among St. John's College undergraduate students and analyze the factors associated with stress. The findings revealed that a significant majority of students experienced moderate to high levels of stress, with 64.4% reporting moderate stress and 21.3% reporting high perceived stress. These results are consistent with the existing literature, which indicates that stress is a prevalent issue among university students globally. Previous studies have highlighted the high prevalence of stress among college students. For instance, Misra et al. (2000) found that college students often experience elevated stress levels due to academic pressures, social adjustments, and lifestyle changes [12]. Similarly, a study reported that stress among college students is a significant concern, impacting their academic performance and overall well-being [1].

The comparison of stress levels across different courses revealed that students in the B.Sc. program had a higher proportion of low stress, whereas B. Com. students reported the highest levels of high perceived stress. Although the differences in stress levels between courses were not statistically significant (p = 0.138), these findings suggest that the nature of the curriculum or course requirements may influence stress levels. Research by Robbins et al. (2004) supports the notion that different academic programs can affect students' stress levels differently [13].

The analysis of sociodemographic factors showed significant associations with gender and area of residence. Female students and those from urban areas were more likely to report high levels of stress. This finding is in line with the studies indicating that female students often experience higher stress levels than their male counterparts [4], and urban environments may present additional stressors, such as increased competition and higher living costs [14]. Other sociodemographic factors, such as parental education and economic status, did not significantly impact stress levels in this study, which contrasts with some literature suggesting that these factors can influence stress [15]. The study highlights the need for targeted interventions to manage stress among students, particularly those in high-stress courses and those from urban areas. Institutions could benefit from implementing stress management programs and providing resources to support students' mental health.

Limitation

One limitation of this study is the reliance on self-reported data, which may introduce response bias, as students might underreport or overreport their stress levels due to social desirability or misinterpretation of the questions. Additionally, the study was conducted at a single college, which limits the generalizability of the findings to other student populations in different educational settings or geographical locations. While adequate for the study's scope, the sample size may still not capture the full range of stress experiences among all students. Lastly, the study's cross-sectional design prevents the establishment of causality, meaning that while associations between stress levels and certain variables were identified, it is impossible to determine whether these variables directly cause changes in stress levels.

## Conclusions

The study highlights the prevalence of moderate to high levels of stress among students, with a significant portion experiencing considerable stress. The findings suggest that stress is influenced by certain sociodemographic factors, particularly gender and area of residence, with female students and those from urban areas being more prone to higher stress levels. While the differences in stress levels across various courses were not statistically significant, the overall data underscore the need for targeted stress management interventions within the student population. Addressing these stressors through supportive measures and resources could enhance student well-being and academic performance. Further research, potentially involving a larger and more diverse student sample, is recommended to deepen the understanding of stress determinants and to develop effective strategies for mitigating student stress.
